# Current Status and Trends in Research on Caries Diagnosis: A Bibliometric Analysis

**DOI:** 10.3390/ijerph19095011

**Published:** 2022-04-20

**Authors:** María Melo, José Luis Sanz, Leopoldo Forner, Francisco Javier Rodríguez-Lozano, Julia Guerrero-Gironés

**Affiliations:** 1 Department of Stomatology, Universitat de Valencia, 46010 Valencia, Spain; m.pilar.melo@uv.es (M.M.); forner@uv.es (L.F.); 2 Gerodontology and Special Care Dentistry Unit, Morales Meseguer Hospital, Faculty of Medicine, University of Murcia, 30100 Murcia, Spain; fcojavier@um.es (F.J.R.-L.); julia.guerrero@um.es (J.G.-G.)

**Keywords:** bibliometric, scientometric analysis, caries diagnosis, caries detection, citations

## Abstract

There are a wide variety of devices for the detection and diagnosis of caries from the initial stages. The purpose of this study was to perform a bibliometric study on research regarding caries diagnosis by identifying the contributing researchers, organizations, countries or regions, journals, and to provide an analysis of keyword co-occurrence and co-authorship networks. An advanced search was performed in Web of Science (WOS) Core Collection database, using the terms “caries diagno*” and “caries detect*” in the “topic” field, from 2013 to 2021. Bibliometric parameters were extracted using WOS’s analyze results tools and VOSviewer software. A total of 816 documents were identified. Most of them, (61.3%) are included in “Dentistry Oral Surgery & Medicine” category within WOS. The largest scientific production on the subject is observed between 2018 and 2021, with a total of 344 records. The most productive author is Mendes FM, followed by Braga MM. The journal with the most articles published on caries diagnosis is *Caries Research*, with 55 articles (6.74%). The terms with the highest co-occurrence refer to the validity of diagnostic methods, tools or principles used in diagnosis or general aspects related to caries detection and diagnosis.

## 1. Introduction

Dental caries is the most prevalent chronic disease in the world [1]. Its proper diagnosis is paramount in daily dental practice, currently focused on performing evidence-based non-invasive or minimal intervention treatments, based on respect for dental tissues whenever possible, and aimed at promoting and maintaining dental health [2,3].

There are different methods for the diagnosis of carious lesions. However, none of them has shown the maximum sensitivity and specificity, so research continues to find one that meets the needs of professionals. The International Caries Detection and Assessment System (ICDAS) is a method based on visual inspection [4] which is widely accepted by the scientific community [4,5]. Bitewing radiographs are considered the gold standard for the diagnosis of interproximal caries [6,7,8]. However, different methods based on optical principles such as transillumination [8,9] or fluorescence [10,11] have been developed, along with others based on electrical impedance or conductance [12] to detect early carious lesions as well as remineralization over time [13]. Both the diagnosis of carious lesions in its early stages and the diagnosis of the risk of caries development are a topic of interest, both at the patient’s and the tooth’s level [14].

Bibliometric studies perform a series of analyses that allow to report the scientific production on a topic in a structured way, as well as the impact of individual researchers and research groups on a particular topic through the use of citation metrics, such as the number of citations, production by authors, journals, countries and institutions where research is carried out, etc. [15]. Data obtained through bibliometric analyses allows to identify changes over time in trends in scientific production, dominant areas of research interest, as well as their growth and development. In addition, bibliometric analyses can detect the most productive authors or research groups and present an overview of existing research [16].

In recent years, bibliometric studies have focused on different aspects related to dental caries. For example, Gomes Silva et al., (2021), published a bibliometric study on the recommendations made from American Academy of Pediatric Dentistry on breastfeeding and sugar consumption and oral hygiene in infants for the prevention of dental caries [17] Baldiotti et al., (2021) analyzed the 100 most cited articles in cariology [18], and Patil et al., (2020) analyzed the 100 most cited articles on “childhood caries” [19].

However, to our knowledge, there are no published bibliometric studies on the scientific production regarding caries diagnosis. Accordingly, the purpose of this study was to perform a bibliometric study on research regarding caries diagnosis by identifying the contributing researchers, organizations, countries or regions, journals, and to provide an analysis of keyword co-occurrence and co-authorship networks.

## 2. Materials and Methods

In the development process of the search strategy, the range of years from 2013 to 2021 was selected based on the obsolescence index of the journals included in Journal Citation Reports (JCR) in the category “Dentistry, Oral Surgery and Medicine”, which resulted in an average of 8.59 years. Records published in the aforementioned period were identified in January 2022 from Web of Science (WOS) core collection database, with the following search string: (“caries diagno*” OR “caries detect*”), using the “topic” field, which performs the search in the title, abstract, and keywords. The search was refined by limiting the search to original articles and reviews in the “type of document” section, excluding proceedings papers, meeting abstracts, editorial material, and letters.

Two different tools were used to analyze the data: WOS platform [20] and VOSviewer software (VOSviewer v1.6.14.; Center for Science and Technology Studies, Leiden University, Leiden, The Netherlands) [14].

WOS platform provides the following bibliometric data by means of its “analyze results” tool: WOS category, type of document, publication titles, publisher, funding agencies, authors, groups of authors, authors’ countries/regions, languages, and years of publication, among others. Additionally, the citation count, citation density, and Hirsch index (h-index) can be obtained with WOS’s “citation reports” tool. The latter is defined as the number of papers with a citation count ≥ h, which is a useful index to characterize the scientific impact of a researcher/journal [21].

Parallelly, VOSviewer software is a tool used to illustrate bibliometric networks with an automatic algorithm for the identification of terms and authors. The software analyzes the authors with the highest scientific production on a topic, as well as the working groups and the relationships between them, which are reflected in co-authorship networks. In addition, it allows the visualization of the most used terms in the title, abstract or both, and their relationships (keyword co-occurrence networks). In the present study, the term maps were used to identify the distribution of the most used words in research on caries diagnosis over the last nine years. In the networks, the most strongly related terms are located closer to each other, while those with less correlation are further apart [22]. “Overlay visualization” tool from WOS was used to display the keyword co-occurrence and co-authorship networks distributed by years, so that we can establish, for example, the period of time in which a term has been most used, or an author has published the largest number of publications on the topic.

**Figure 1:**
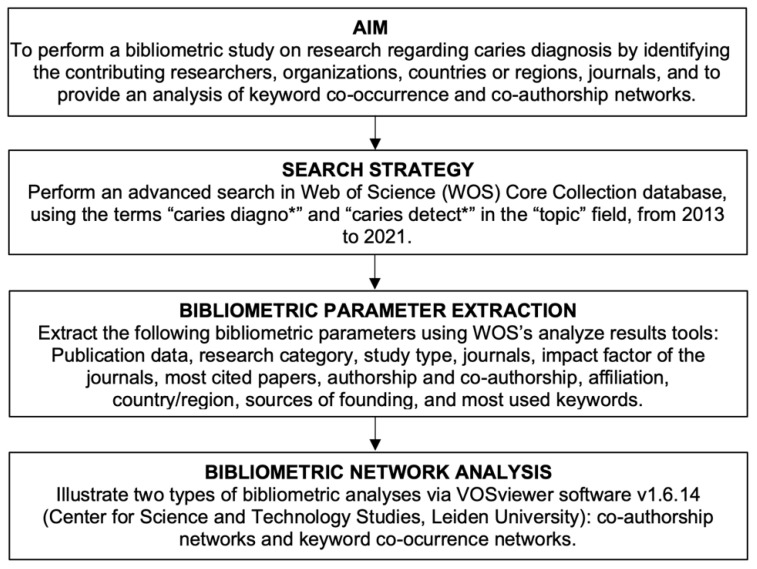
Study protocol.

In the present analysis, the following variables were evaluated: Publication data, research category, journals, impact factor of the journals, most cited papers, authorship and co-authorship, affiliation, country/region, sources of founding, and most used keywords. The protocol for the present bibliometric study is illustrated in Figure 1.

## 3. Results

The search performed in WOS Core Collection within the period of January 2013 to December 2021, after refining the search following the inclusion criteria, resulted in a total of 816 records.

**Figure 2:**
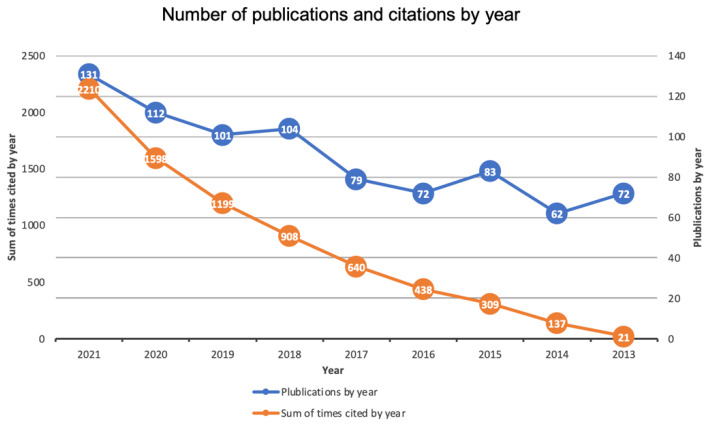
Temporal evolution of the number of publications and total number of citations.

When analyzing the temporal evolution of the scientific production, an incremental trend was observed. The maximum number of publications appeared between 2018 to 2021 (344 articles). The distribution of publications and total number of citations by year of publication is shown in Figure 2.

**Figure 3:**
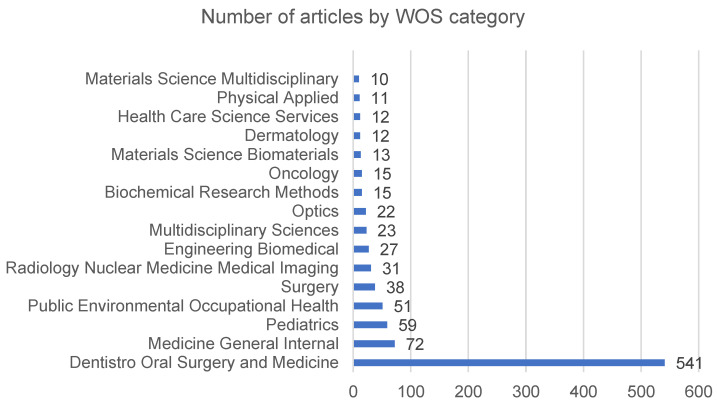
Research areas of the journals with the largest number of publications on the subject under study. The vertical axis shows the categories, while the horizontal axis shows the number of publications.

Among the research categories provided by WOS, those containing 10 or more publications are presented in Figure 3. The category with the highest number of publications is “Dentistry, Oral Surgery & Medicine”, with 543 records (61.30%), followed by “General Internal Medicine”, “Pediatrics” and “Public Environmental Occupational Health” at a substantial distance from the first.

Regarding the distribution of publications by study type, the following order was observed: in vitro studies (234), cross-sectional studies (207), quasi-experimental studies (150), randomized controlled trials (60), cohort studies (56), narrative reviews (51), meta-analysis/systematic reviews (39), case report/series (19).

**Figure 4:**
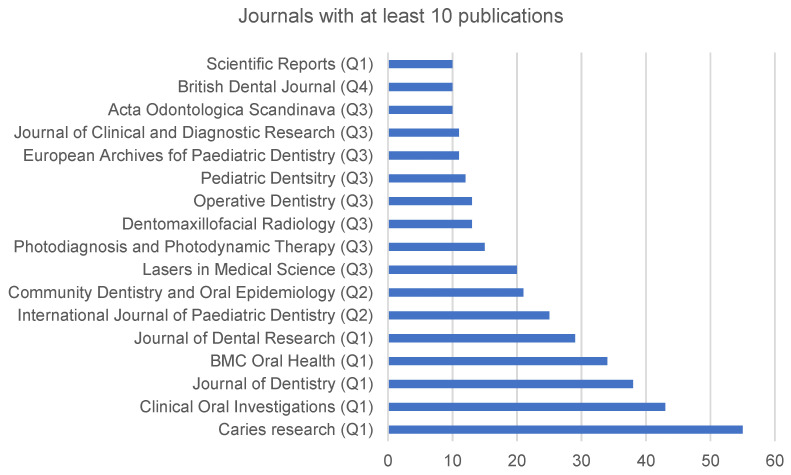
Journals with at least 10 publications with the topic “caries diagno*” or “caries detect*”.

From the 25 journals in which the records are distributed, those with 10 or more publications are shown in Figure 4. The journals with the highest number of records are *Caries Research* with 55 publications (6.74%), and Clinical Oral Investigations with 43 articles (5.27%). Regarding the type of publications, 753 (92.72%) are original articles, 63 (7.21%) are reviews and 18 (2.32%) are early access papers. A total of 375 articles related to caries diagnosis were identified as open access publications. Impact factor is a measure of the importance of a scientific publication. It is calculated over a two-year period as the quotient of the number of times an article has been cited in the previous two years divided by the number of articles published in the journal in those two years [23]. From the 10 journals with the highest number of publications related to caries diagnosis, five of them are in the first quartile (Q1), two in Q2, and one in Q3 within the category “Dentistry, Oral Surgery & Medicine”. Journal quartiles are defined as Q1 (0.0 < Z ≤ 0.2), Q2 (0.25 < Z ≤ 0.5), Q3 (0.5 < Z ≤ 0.75), and Q4 (0.75 < Z), where Z is defined as the journal rank in a specific category divided by the number of journals in the specific category. Therefore, the highest ranked journals in a category belong to Q1, and the lowest ranked journals belong to Q4. The impact factor of these journals ranges from 6.116 for *Journal of Dental Research* to 2.419 for *Dento Maxillofacial Radiology*. The other two journals that do not belong to this category are *Lasers in Medical Science*, which is in Q2 in the two areas in which it is included (“Dermatology and Surgery” and “Photodiagnosis”) and *Photodynamic Therapy*, which is in Q3 in the category “Oncology”. The number of times the articles have been cited was 7459. If self-citations are eliminated, the number reduces to 5417, with an average number of citations per article of 9.14, and an h-index of 37.

The 20 most cited articles, with their authors, years of publication, total number of citations, mean number of citations per year, the journal in which they are published, type of article, and aspects studied, are presented in Table 1. 15 of the 20 articles are published in journals in “Dentistry, Oral Surgery & Medicine” category, among which 7 are in first quartile journals (three in *Journal of Dental Research*, three in *Journal of Dentistry*, and one in *Clinical Oral Investigations*), six in second quartile journals, (one in *International Journal of Paediatric Dentistry*, two in *Community Dentistry* and *Oral Epidemiology*, two in *BMC Oral Health* and one in *Oral Surgery Oral Medicine Oral Pathology Oral Radiology*) and one in the fourth quartile journal *Quintessence International*. The most cited article is that of Pitts et al., (2013) [24], which refers to the use of the ICDAS system for the categorization of carious lesions, combined with the Classification and Management System (ICCMS) for caries management, ranked 21/82 that year. The second place was taken by an article proposing an algorithm-based system for radiological caries diagnosis, published by Wang CW et al. in 2016 [25]. There are 11 other articles related to complementary methods for caries diagnosis, based on radiology, different types of light sources, algorithms, and convolutional neural networks. Two are epidemiological studies by Prasai Dixit et al., (2013) [26], published in *BMC Oral Health* which ranked 48/82 (Q3), and that of Nobile CG (2014) [27], published in *BMC Public Health* under the category “Medicine”, with an impact factor of 2.696 in the year of publication.

**Figure 5:**
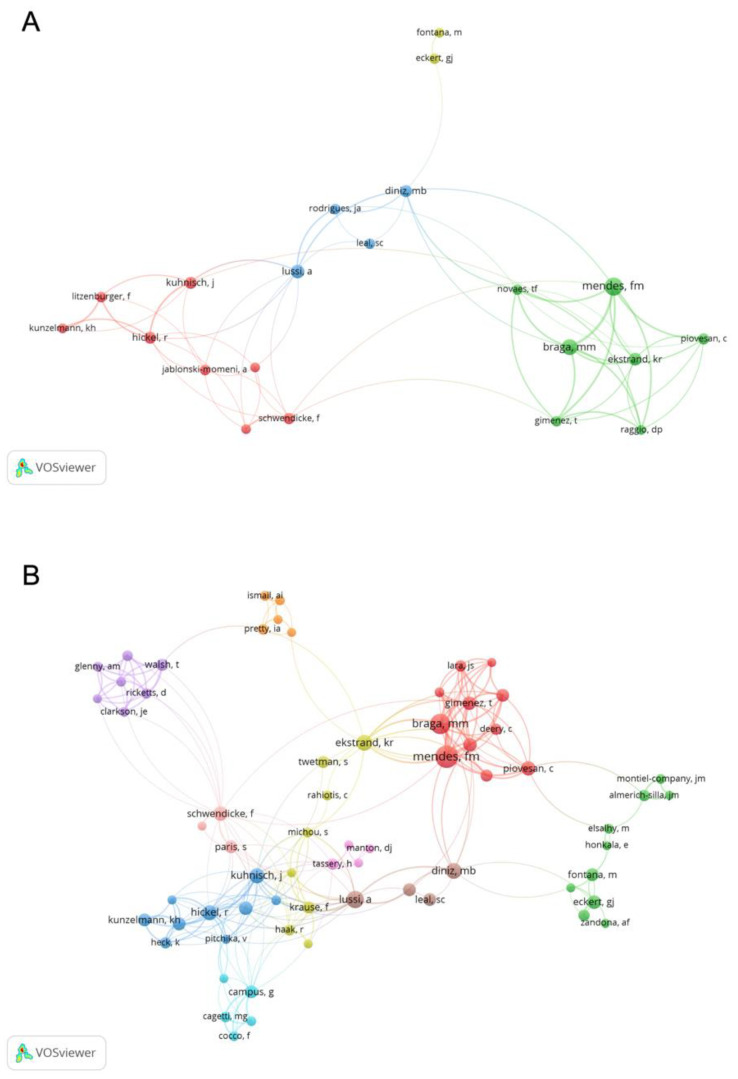
(**A**) Authors with at least ten publications on caries diagnosis and the collaborative relationship between them. (**B**) Authors with at least five publications on caries diagnosis and collaborative relationship between them. Bubble size indicates number of documents. Link length indicates closeness of collaboration.

Regarding authors, the three most productive were: Mendes FM with 32 publications, followed by Braga MM with 25, and Lussi A with 19. The co-authorship network illustrated by VOSviewer software is based on the most productive authors. Authors with at least ten publications on the subject are grouped in 5 nodes, although those in the “top 5” are part of only two groups: Mendes FM, Braga MM, Ekstrand KR in one of them, and Lussi A and Diniz MB are included in the other one (Figure 5A). If we reduce the minimum number of publications to five, the number of authors meeting this requirement increases, and the number of working groups (co-authorship networks) also increases to ten (Figure 5B).

**Figure 6:**
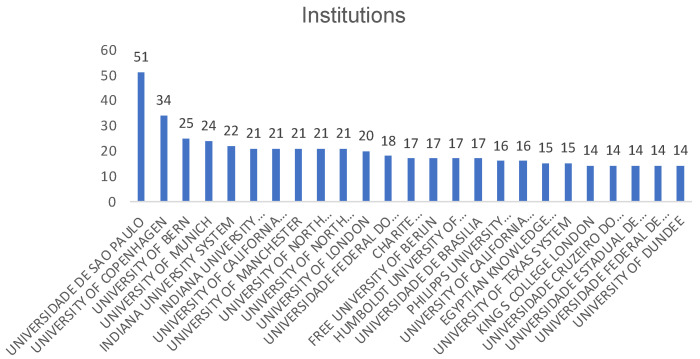
Institutions with the highest number of publications.

The affiliation of the researchers is presented in Figure 6: the University of Sao Paulo in Brazil stands out (51 publications), where the most productive author (Mendes FM) is located, followed by the University of Copenhagen (34 publications). Another 22 institutions range between 25 and 14 publications.

**Figure 7:**
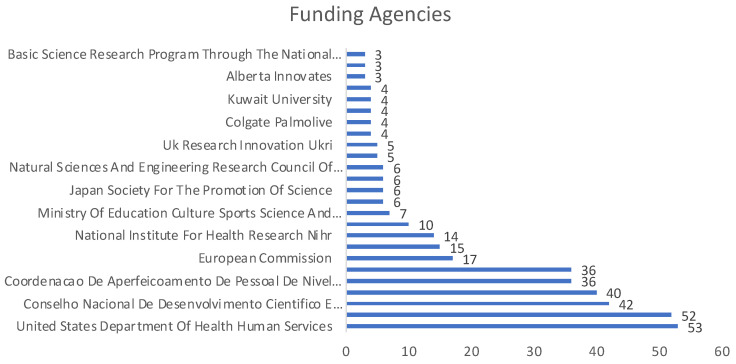
Entities that finance the largest number of publications.

Regarding the sources of funding of publications, Figure 7 shows the 25 most representative entities, from which the following funded between 36 and 51 publications: Fundaçao de Amparo a Pesquisa do Estado de Sao Paulo (FAPESP), Coordenaçao de aperfeicoamento de Pessoal de Niivel Superior (CAPES), Conselho Nacional de Desenvolvimento Cientifico e Tecnologico (CNPQ), National Institute of Dental Craniofacial Research (NIH), National Institutes Of Health (NIH), and United States Department Of Health Human Services (HHS).

**Figure 8:**
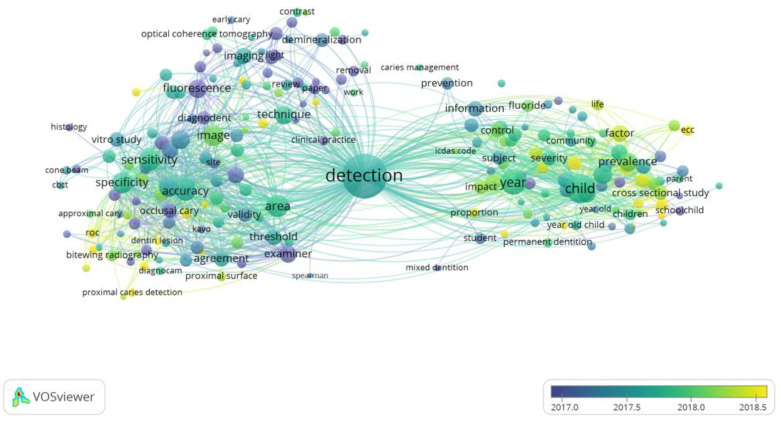
Map of terms appearing at least ten times. Bubble size indicates occurring frequency of terms as keywords in publications. The color of the bubble indicates the period in which terms are most used, choosing the time period where the greatest chromatic diversity is found.

Nineteen words were considered as highly relevant (more than 100 occurrences) in the present search. These are, in order of relevance: “specificity”, “sensitivity”, “accuracy”, “child”, “fluorescence”, “performance”, “detection”, “year”, “caries detection”, “area”, “group”, “international caries detection”, “assessment system”, “dental caries”, “ICDAS”, “diagnosis”, “study”, “data” and “difference”. The map of terms (Figure 8) revealed that words related to the parameters for determining the validity of diagnostic methods (“sensitivity”, “specificity”, or “agreement”, among others) had a substantial presence. On the other hand, words referring to different tools or principles used in the diagnosis of carious lesions (such as “fluorescence”, “optical coherence tomography”, “QLF”, “imaging” or “bitewing radiography”) also show an important relevance. Another group of words refers to general aspects related to caries detection and diagnosis (“prevention”, “risk assessment”, “year” or “child” among others). No particular upward or downward temporal evolution or trend was observed for any of the keywords in the overlay-visualization keyword co-occurrence network.

## 4. Discussion

Dental caries is a disease of multifactorial etiology, mediated by biofilms which are modulated by diet [28]. Social, behavioral, and environmental factors significantly influence caries risk [29]. It is a disease with a high prevalence, being a worldwide public health problem and its control a global challenge [30].

Caries risk diagnosis and early identification of caries lesions is a challenge for daily clinical practice [8,13]. This study confirms that the diagnosis of caries is a current issue since the number of articles published in this regard has increased from 2013 to 2021, with an upward trend during this period. The development of new techniques and methods for carious lesion diagnosis plays an important role in daily clinical practice.

The sources of bibliographic information can be divided into two main groups according to the paradigm of evidence-based medicine: primary sources of information (where it is necessary to make a critical appraisal of the articles to determine their scientific validity and clinical application) and secondary sources of information (which usually involve a critical appraisal of the documents) [31]. The type of publication can influence the number of citations it receives. In general, in scientific disciplines, review articles are more often cited than other article types [32]. Systematic reviews and meta-analyses are useful study designs, as they may guide clinical practice and health policies [33]. These studies do not involve high costs and have a high probability of being cited in other papers [34].

In the present study, the most cited articles include in vitro, in vivo, cross-sectional studies and literature reviews. The one in first place [24] refers to ICDAS and ICCMS, used as an international consensus and now is being adapted for post-pandemic use in the ‘Caries OUT’ study [35]. The use of intraoral radiographs for the diagnosis of interproximal caries is currently considered the gold standard [8]. Among the articles in the “top ten”, two refer to radiology for caries diagnosis [25,36]. The first one, published in 2016, proposes a model for the objective evaluation of bitewing radiographs using automatic algorithms. The second is a systematic review and meta-analysis, which evaluates the usefulness of bitewing radiography to assess lesions of different locations and extension, pointing out that this scan is of great utility in the diagnosis of interproximal and occlusal caries when they affect dentin. However, its sensitivity is limited in the diagnosis of incipient lesions.

The most widely used method for caries diagnosis is still visual inspection [10]. The incorporation of indices that allow the identification of minimal changes in the enamel helps in the early detection of lesions, however, the meta-analysis by Gimenez T et al., (2015) [37], which ranks fifth among the most cited in the present review, highlights the heterogeneity of the studies and the need to implement other methods. Among these methods are, quantitative light-induced fluorescence (QLF), DIAGNOdent (DD), fiber optic transillumination (FOTI) and electrical conductance (EC). These methods also make it possible to monitor the evolution of lesions over time after the application of biomineralization procedures [1]. The interest in these new methods has become evident in the present bibliometric study: In addition to the aforementioned review by Gomez (2015), which is in eighth place among the most cited articles, two articles on the application of near-infrared light transillumination in the early diagnosis of caries lesions are found among the 20 most cited articles [38,39,40,41]. The keyword co-occurrence analysis can also give insight into the popularity of caries diagnosis methods. As observed in Figure 8, the terms “bitewing radiography”, “fluorescence”, “ICDAS code” and “Diagnodent” appear among the most used terms. Based on the results, from the keyword co-occurrence analysis summary of the main methods for caries diagnosis which appeared in the included studies is presented in Table 1.

The impact index values of the most important scientific journals are published on Journal Citation Reports [42]. This impact index has a number of limitations, such as the fact that it can be very different depending on the selected area [43]. In some cases, the journal with the highest impact index in one area may have a lower impact index than the journal with the lowest impact index in a different area. This is the case of two of the journals that appear with the highest number of publications in our study: the first, Caries Research (with 55 articles) belongs to the category “Dentistry, Oral Surgery & Medicine” and has an impact factor in 2020 of 4.056, while the first journal in the ranking (Journal of Clinical Periodontology) has an impact factor of 8.728. However, the 17th ranked journal (Scientific Reports, with an impact factor of 4.13) is in the “Multidiciplinary Sciences category”, in which the journal with the highest impact factor is Nature with 49.962. There is a standardized index called Source Normalised Impact per Paper (SNIP) that tries to compensate for these differences by weighting the number of citations of an article according to the total number of citations in that particular area [44]. Standardization in scientific journals is a necessary condition since without it the universal dissemination of scientific knowledge would not be possible [45]. With the evolution of research, various guidelines have been proposed to determine and increase the quality of published articles, whether they are laboratory studies, epidemiological, clinical or reviews [46,47,48].

*Caries Research* is the journal with the highest number of publications in this topic, and it is also—together with the *Journal of Dental Research*—the journal with the highest number of articles in the top 100 journals in the field [18]. The present study also found that some papers were published in prestigious journals in basic and health sciences such as *Lasers in Medical Sciences*, *Photodiagnosis and Photodynamic Therapy* or *Scientific Reports*, which indicates the importance of caries lesion detection in other fields and the interest of other disciplines in creating links with dentistry. This trend is also reflected when analyzing WOS categories, which is worth remembering as they have their role as reference standards for bibliometric evaluation [49].

Articles published in open access represent less than 50% of the total number of publications in this study [50], which allows them to gain visibility and therefore more citations from other authors. Open access to scientific articles is now a requirement of most research councils and project funding programs [51].

The analysis of scientific articles produced by different groups of authors helps to identify and characterize research groups and collaborations among them. Scientific collaboration can be expressed in different forms, and one way to identify a collaboration process in Science is the co-authorship of research papers [52]. Authors’ network positions in co-authorship networks influence the performance and formation of scientific collaborations [53,54]. The identified authorship nodes are grouped under the same institution, although logically there are collaborations with other nodes. This collaboration between authors and organizations is useful to increase the number of publications. This information is of interest to researchers, students and professionals, who can identify leading researchers in the field and research organizations [55] who play an important role in disseminating information on caries detection and diagnosis.

Keywords play a fundamental role in the classification of published scientific articles [56]. In this study, the most relevant keywords (specificity and sensitivity) are not those with the highest number of occurrences. In the same line, it should be noted that using the automatic term identification function of the VOS software, some of the most relevant terms refer to general concepts such as “child” or “year”. In case you want to remove those terms, software can remove general terms by calculating the relevance score [57,58], although they can also be removed manually. The most relevant principle is “fluorescence”, followed by “ICDAS”. The rest of the terms that appear at least ten times and are represented in Figure 7 of the results (optical coherence, CBCT, or bitewing, among others) do not reach an important relevance, although they do appear on numerous occasions in the articles included in this study. The occurrence is the presence, frequency, and proximity of similar keywords [59]. However, relevance indicates the importance of that word in the field in question. It should be noted that the VOS software selects 60% of the most relevant terms used in at least ten documents to focus the information on those that will give the reader more information. Thus, it can be stated that the aforementioned methods or systems are the ones that have aroused the most interest among researchers in the last nine years. The use of appropriate keywords by both the authors of the articles and the researchers seeking information on the subject are key to the successful dissemination of information and research [60]. It is therefore advisable to include terms that refer to specific operating principles in this case.

Clinicians and researchers possess lots of information to perform the correct management of carious lesions, according to the evidence [3,14]. It is therefore convenient to know the sources where to look for that information or the most used descriptors, among other aspects [61], as well as the choice of keywords [62]. Choosing an appropriate title is important for precise coding in programs and electronic search engines, thus influencing the article’s impact on the scientific community [63,64].

## 5. Conclusions

Caries diagnosis is a subject that involves a substantial scientific production, which is exponentially increasing. Publications are distributed in different categories, among which some that do not belong to the “Dentistry” area are gaining relevance. The keywords that have been found to be most relevant refer to the validity of diagnostic methods, tools or principles used in diagnosis or general aspects related to the detection and diagnosis of caries. Parallelly, in vitro studies are the most common study type in the field of caries diagnosis. The present bibliometric analysis sheds light on the need for clinicians to assess the type, quantity, and quality of the available evidence before implementing new diagnostic tools or methods into daily clinical practice.

## Figures and Tables

**Table 1:** Assessed methods for caries diagnosis.

Methods for Caries Diagnosis	Characteristics and/or Examples
ICDAS II (International Caries Detection and Assessment System)	Coded classification (0–6) based on visual inspection
Radiographic images	Intraoral bitewing radiographic images (conventional or digital)
Transillumination-based methods	Fiber optic transillumination (FOTI) Digital Imaging Fiber Optic TransIllumination (DIFOTI) Near-Infrared Light-Transillumination (NILT) (780 nm)
Fluorescence-based methods	Diagnodent^®^ (655 nm), Vistaproof^®^ (405 nm), QLF^®^ (488 nm)
Fluorescence and infrared thermography-based methods	Canary System^®^
Methods based in the analysis of electric conductance changes	Caries Monitor^®^, Cariometer^®^, CarieScan^®^
Experimental methods	Optical coherence tomography, Terahertz images, Raman polarized spectroscopy, Multiphoton microscope

## Data Availability

The data presented in this study are available on request from the corresponding author.
